# The study of the *Oxytropis kansuensis*-induced apoptotic pathway in the cerebrum of SD rats

**DOI:** 10.1186/1746-6148-9-217

**Published:** 2013-10-22

**Authors:** Hao Lu, Liang Zhang, Shan-shan Wang, Wen-long Wang, Bao-yu Zhao

**Affiliations:** 1 College of Veterinary Medicine, Northwest A&F University, Yangling, Shaanxi 712100, People’s Republic of China

**Keywords:** *Oxytropis kansuensis*, Swainsonine, SD rats, Cerebrum, Apoptotic pathway

## Abstract

**Background:**

Locoweeds cause significant livestock poisoning and economic loss all over the world. Animals can develop locoism, a chronic neurological disease, after grazing on locoweeds. *Oxytropis kansuensis* is a variety of locoweed that contains swainsonine as its main toxic ingredient. The purpose of this study was to investigate the apoptotic pathway induced in the cerebrum by swainsonine.

**Results:**

Twenty-four Sprague-Dawley rats were randomly divided into four groups (experimental groups I, II, III and a control group) and 6 SD rats of each group were feed in 3 cages separately. Rats were penned as groups and fed with feeds containing 15% (SW content 0.03‰), 30% (SW content 0.06‰), or 45% (SW content 0.09‰) *O*. *kansuensis* for experimental groups I, II, and III, respectively, or complete feed in the case of the control group. One hundred and nineteen days after poisoning, and all rats showed neurological disorders at different degrees, which were considered to be successful established a chronic poisoning model of *O. kansuensis*. rats were sacrificed and the expression of Fas, FasL, Bcl-2, Bax as well as cleaved caspase-3, -8 and -9 proteins in brain tissues were detected by Western blot. The results showed that SW treatment up-regulated Fas and Fas ligand (FasL) (P < 0.05), and that there was an increase in Bax and a decrease in Bcl-2 protein (P < 0.01). Moreover, SW treatment significantly increases the activation of caspase-3, 8 and -9, the key effectors in apoptosis pathway (P < 0.01).

**Conclusion:**

Our data suggest that SW induces apoptosis in cells of the brain through death receptor and mitochondria-mediated, caspase-dependent apoptotic pathways in the brain tissue of SD rats.

## Background

Locoweeds are a general term for toxic plants of the genera *Astragalus* and *Oxytropis*, and are one of the toxic plants causing the most damage to husbandry in the grassland across the world. They possess wider geographical distribution from the Great Plains to the Rocky Mountains, and they are worldwide spread, such as in the United States [1], Australia [2], Mexico [3], Brazil [4], and China [5]. *Oxytropis kansuensis*, one of the locoweeds from the genus *Oxytropis*, is found in Qinghai, Gansu, Ningxia, Sichuan, northwestern Yunnan, and paramos regions in Tibet [6]. Because of its luxuriant leaves, wide-spread roots and strong stress resistance, huge numbers of livestock get poisoned and even die in grassland containing *Oxytropis kansuensis* every year, causing huge losses to local herdsmen and typical ecological-economic disease [7]. Poisoned animals show clinical signs characterized by nerve functional disturbance symptoms like depression, diminished response, ataxia, abnormal behavior, emaciation and decline in immune function [8,9].

**Figure 1:**
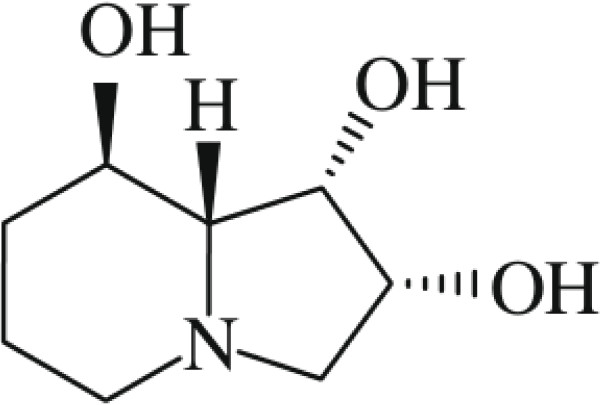
The structure of swainsonine.

The main toxic ingredient in locoweeds is swainsonine (SW) [10], an indolizidine alkaloid whose chemical name is 1, 2, 8- trihydroxy-indolizidine alkaloid (Figure 1). SW was first isolated from *Swainsona canescen* by Colegate et al. [11]; since then, large amounts of researches has focused on the bioactivity of SW, finding that the structure of SW is similar to the mannose cation formed by the hydrolysis of mannosidase. SW has high affinity to α-mannosidase and, as a result, can inhibit the enzyme. This inhibition induces abnormal of glycoprotein processing and cellular vacuolation [12], especially in nerve cells that is caused by the accumulation of oligosaccharides in the lysosome [13,14].

Apoptosis is a tightly controlled physiological process that plays a critical role in developmental modeling, homeostasis maintenance, immune repertoires, and clearance of infected or transformed cells [15]. Apoptosis can be triggered by various extracellular and intracellular stimuli via either an extrinsic or intrinsic pathway in different cells [16]. The extrinsic pathway is initiated by cell surface receptors, while the intrinsic pathway is initiated by a mitochondria mediated death signaling cascade [16]. To date, SW has been reported to induce the apoptosis of the gastric cancer cell SGC-7901, C6 glioma cells, human lung cancer cell A549, etc. Some studies have been conducted examining the apoptosis pathway induced by SW in A549 cells [17-19]. However, the mechanism SW-mediated neurotoxicity has not been rigorously explored, and apoptosis in the brain induced by SW and its associated pathways have yet to be discovered. Thus, the objective of this study was to determine the effects of SW on the expression of Fas, FasL, Bcl-2, Bax and cleaved caspase-3, -8 and -9 in the brains of SD rats and to determine which apoptosis pathway is induced by SW. These results will inform future research on the mechanisms underlying the toxicity of *O. kansuensis* as well as other locoweeds.

## Results

### *O. kansuensis* activates caspase-8, -9 and -3

**Figure 2:**
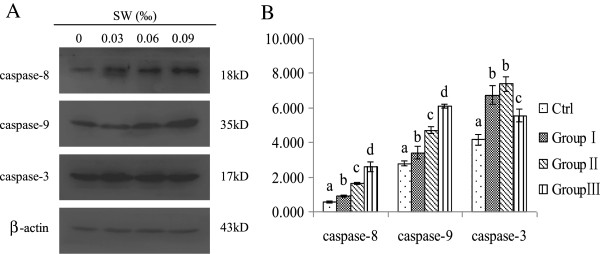
**Effects of *O. kansuensis *treatment on caspase activation in the cerebrum. (A)** Western blot analysis for caspase activation in *O. kansuensis* treated rats at different doses for 119 d. (Groups I (*O. kansuensis* 15%, SW content 0.03‰), Group II (*O. kansuensis* 30%, SW content 0.06‰), Group III (*O. kansuensis* 45%, SW content 0.09‰)). The molecular weight (kDa) of protein size standards is shown on the right hand side. **(B)** Quaint One was used to assay for quantitate protein levels of cleaved caspase-3, -8, and -9. Values are shown as means ± SEM. The data shown are representative of three independent experiments. Different letters indicates significant difference (P < 0.05), while the same letters were not significantly different.

To gain insight into the underlying mechanism of *O. kansuensis*-induced apoptosis, we measured the activation of caspases in the brain cells of these rats. Protein levels of caspase-8, -9 and -3 were measured by Western blot. Upon *O. kansuensis* treatment, cleaved caspase-8, cleaved caspase-9 and cleaved caspase-3 levels increased with increased toxic doses, cleaved caspase-8 and cleaved caspase-9 showed a dosed-dependent increase (Figure 2). These data suggest that both death receptor pathway and mitochondrial pathways may contribute to caspase-3 activation in *O. kansuensis*-induced apoptosis.

### *O. kansuensis* induces apoptosis through the Fas/FasL-dependent pathway

**Figure 3:**
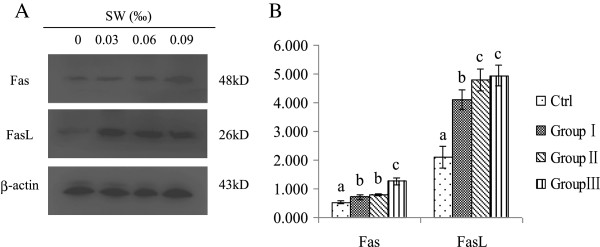
**Effect of *O. kansuensis *treatment on Fas and FasL expression in the cerebrum. (A)** SD rats were treated with *O. kansuensis* at different doses for 119 d (Groups I (*O*. *kansuensis* 15%, SW content 0.03‰), Group II (*O*. *kansuensis* 30%, SW content 0.06‰), Group III (*O*. *kansuensis* 45%, SW content 0.09‰)); β-actin was used as an internal loading control. Protein levels of Fas and FasL were analyzed by Western blot. The molecular weight (kDa) of protein size standards is shown on the right hand side. **(B)** Quaint One was used to quantitate protein levels of Fas and FasL. The results are mean ± SD and representative of three independent experiments. Different letters indicates a significant difference (P < 0.05), while the same letters were not significantly different.

Caspase-8 is the initiator caspase for the caspase cascades activated by death receptor pathways [20]. The activation of caspase-8 suggested that Fas and FasL may be involved in *O. kansuensis*-induced apoptosis. Therefore, we investigated the expression of Fas and FasL in the brain cells of SD rats treated with *O. kansuensis*. As shown in Figure 3, the protein levels of Fas and FasL were increased in a dose-dependent manner.

### *O. kansuensis* regulates the expression of Bcl-2 family proteins

**Figure 4:**
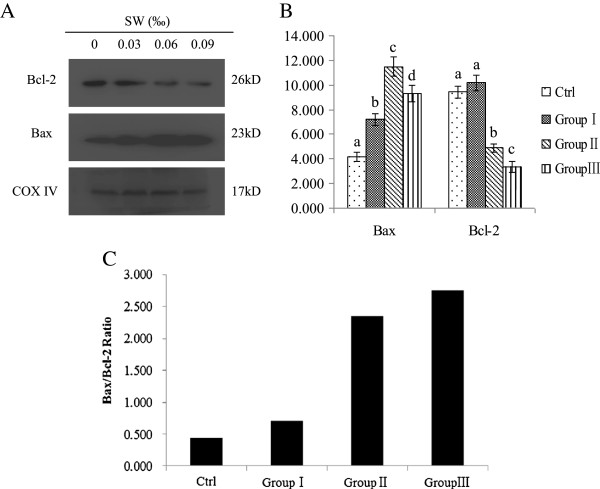
**Effect of *O. kansuensis *treatment on the expression of Bcl-2 family proteins in the cerebrum. (A)** SD rats were treated with *O. kansuensis* at different doses for 119 d (Groups I (*O*. *kansuensis* 15%, SW content 0.03‰), Group II (*O*. *kansuensis* 30%, SW content 0.06‰), Group III (*O*. *kansuensis* 45%, SW content 0.09‰). Protein levels of Bcl-2 and Bax were analyzed by Western blot. The molecular weight (kDa) of protein size standards is shown on the right hand side. **(B)** Quaint One was used to quantitate protein levels of Bcl-2 and Bax. Values are shown as means ± SEM, The data shown are representative of three independent experiments. Different letters indicates a significant difference (P < 0.05), while the same letters were not significantly different. **(C)** Date from **(A, B)** was used to evaluate the effect of *O. kansuensis* on the ratio of Bax/Bcl-2.

The expression of Bcl-2 and Bax were detected by Western blot. Protein levels of Bax were up-regulated, while, conversely, the protein levels of Bcl-2 were significantly decreased (Figure 4A). As shown in Figure 4B, *O. kansuensis* resulted in a dose-dependent increase in the ratio of Bax/Bcl-2. These results suggested that Bax up-regulation and Bcl-2 down-regulation may play important roles in *O. kansuensis*-induced apoptosis of brain cells in SD rats.

## Discussion

The indolizidin alkaloid SW is present in *O. kansuensis*, and has been shown to have neurotoxic effects. Previous studies have reported that the poisoning of livestock by locoweeds caused neurons in the cerebral cortex, basal ganglia, thalamus, mid-brain, hippocampus, cerebellum, medulla, and spinal cord to undergo cytoplasmic vacuolar degeneration [21]. Colodel et al. reported that *Swainsona*, *Oxytropis*, *Astragalus*, and Ipomoea poisonings caused multiple cytoplasmic vacuoles in acinar pancreatic cells, hepatocytes, and renal tubular cells, especially in neurons, and led to lesions of the central nervous system [22]. In the present study, we investigated the mechanisms of *O. kansuensis*-induced apoptosis of brain cells in SD rats. These data demonstrated that SW induced apoptosis in the cerebrum via the Fas/FasL and mitochondria-mediated, caspase-dependent apoptotic pathways.

Many previous reports have indicated that numerous neurogenic diseases were related to the apoptosis of neurons [23-25]. Sun et al. reported that SW could induce SGC-7901 cell apoptosis by inhibiting the gene p53 and decreasing the expression of Bcl-2, increasing the apoptotic trigger gene c-myc and loading [Ca^2+^]_I_[17]; they subsequently demonstrated that the mechanisms of SW-induced apoptosis may be related to the expression of apoptosis-related genes and overloading-[Ca^2+^]_i_-induced endoplasmic reticulum stress [18]. Li et al. reported that SW treatment up-regulated Bax, down-regulated Bcl-2 expression, increased the rate of Bax/Bcl-2, and activated the mitochondria-mediated, caspase-dependent apoptotic pathway *in vitro and in vivo*. A549 cells apoptosis occurred in a concentration- and time-dependent manner [19].

Apoptosis programs a cell to actively commit suicide as the results of activation of dedicated intracellular program; the death receptors (extrinsic) and mitochondrial (intrinsic) pathways are the major signaling cascades that lead to apoptosis. The death receptor pathway involves death receptors from the tumor necrosis factor receptor family such as Fas (CD95), TNFαR, DR3, DR4 and DR5. In the death receptors pathway, ligands of the death receptor initiate signaling via receptor oligomerization, which results in the recruitment of specialized adaptor proteins and the activation of caspase-8 [26]. The mitochondrial pathway is dependent on the formation of lipidic pores, which significantly affected the release of cytochrome *c* from the inner membrane of the mitochondria to the cytosol. Holocytochrome c induces Apaf-1 oligomerization, leading to the activation of caspase-9. Caspase-8 and -9 are both initiator caspases and activate downstream effector caspases that are essential for the direct demolition of cellular structures and DNA fragmentation associated with apoptosis [27-29].

Caspases involved in apoptosis can be divided into two functional subgroups based on their roles. Initiators (caspase-2, -8, -9 and -10) are responsible for initiating the activation of caspase cascades for different apoptotic pathways. Effector caspases (caspase-3, -6 and -7) are responsible for demolition of the cell during apoptosis [20]. In this study, we observed that SW-induced apoptosis activated caspase-8, -9 and -3, which suggested that the death receptor-mediated caspase-8 pathway and the mitochondrial-mediated caspase-9 pathway may be responsible for SW-induced apoptosis.

Fas is one of the death receptors; binding of FasL induces Fas trimerization, which recruits caspase-8 via the adaptor protein Fas-associated death domain protein (FADD). Then, caspase-8 oligomerizes and is activated through autocatalysis. Activated caspase-8 triggers the execution phase of apoptosis via the activation of the downstream effector caspase-3 [20,26,30-32]. This study revealed that the SW-induced apoptosis resulted in increased expression of Fas and FasL, followed by activation of caspase-8 and -3. These data demonstrated that caspase-8 activation in SW-induced apoptosis can be mediated by Fas/FasL interaction.

Cytochrome *c* is one of a host of pro-death molecules residing within mitochondria and is a universal feature of apoptosis. Previous studies have demonstrated that the Bcl-2 family is intimately involved in the regulation of cytochrome *c* release into the cytosol [33]. Bcl-2 family proteins include both pro- and anti-apoptotic members. The pro-apoptotic homolog Bax is located in the cytosol, and it can interact with the anti-apoptotic protein Bcl-2. In response to apoptotic signals, Bax translocates to the mitochondria and inserts into the outer mitochondrial membrane, heterodimerizing with Bcl-2 to abrogate Bcl-2’s inhibition of apoptosis by promoting the release of cytochrome *c* into cytosol [34]. Therefore, the ratio of Bax/Bcl-2 sets the threshold of susceptibility to apoptosis for the mitochondrial pathway [20,31,32,35]. Our results showed that SW treatment up-regulated Bax and down-regulated Bcl-2 expression, increasing the ratio of Bax/Bcl-2 which likely resulted in brain cell apoptosis.

## Conclusions

**Figure 5:**
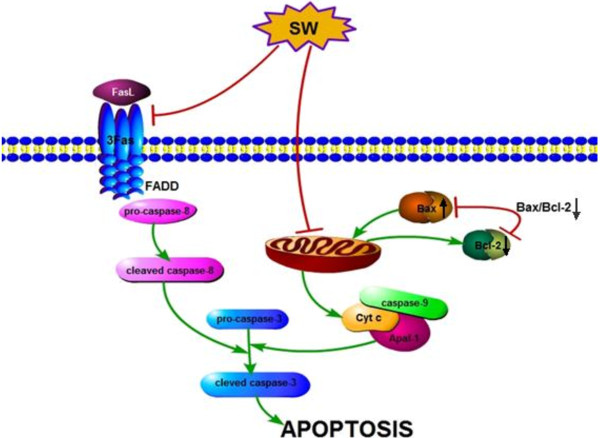
**Schematic representation of the apoptotic pathway in *O. kansuensis*-induced SD rat brain cells.** Feeding with *O. kansuensis* Bunge induces apoptosis via the activation of Fas/FasL-mediated pathway and the initiator caspase, caspase-8. Bcl-2 was down-regulated and Bax was up-regulated, activating the mitochondria-mediated apoptotic pathway and causing the activation of caspase-9. Finally, the activation of caspase-8 and -9 activates caspase-3, leading to apoptosis.

Our results demonstrate that SW induces apoptosis through the activation of Fas/FasL-mediated caspase-8 and mitochondria-dependent caspase-9 pathways. These data provided a new foundation to further explore the underlying mechanisms of *O. kansuensis*-induced apoptosis. Based on these findings, a schematic model of possible SW-induced apoptotic mechanisms in brain cells is shown in Figure 5. However, it is probable that we are far from unraveling the complete mechanisms of SW-induced apoptosis, with mechanisms involving p53, AIF, Smac/DIABLO, and endoplasmic reticulum stress potentially playing a significant role.

## Methods

### Plant materials

The aerial portion of *O. kansuensis* was collected in Huangzhong County (36°29.286'N; 101°41.499'E), Qinghai Province in August 2008. The plants were preserved in a shady place after air-drying and comminution. SW was extracted by TCL and GC-MS mehods [36], SW content was determined by gas chromatography as 0.021% of the total plants content. The plant was identified by the Institute of Botany (Life Science College, Northwest A&F University).

### Animals and animal feed

The animals and protocols used in this study were approved by the Animal Care Committee of Xi’an Jiaotong University. SD rats were purchased from the Experimental Animal Center of College of Medicine, Xi’an Jiaotong University (Xi’an, China). The aerial portion of *O. kansuensis* was completely ground, then sifted through a 200 mesh screen. The resulting *O. kansuensis* grass meal was mixed with whole feed (25% flour, 28% cornmeal, 20% soya-bean cake, 10% wheat bran, 10% fish meal, 1% vegetable oil, 1% yeast powder, 2% bone meal, 1% cooking salt, 1% cod-liver oil, 0.9% mineral additive, 0.1% vitamin additive) [37] to obtain 15%, 30%, and 45% grass content feed containing 0.03‰, 0.06‰, and 0.09‰ SW, respectively [38]. Water was then added to this mixture and stirred into 1-3 cm^3^ diced mixed rations and dried for preservation.

### Chemical reagents and apparatuses

Antibodies purchased from Abcam were: anti-CD95 (ab82419), anti-Fas ligand (ab15285), anti-Bax (ab7977), anti-Bcl-2 (ab7973), anti-caspase-3 (ab32351), anti-caspase-8 (ab25901), anti-caspase-9 (ab32539). Anti-β-actin (BA2305) was purchased from Wuhan Boster Biological Technology, Ltd; peroxidase-conjugated AffiniPure Rabbit anti-goat IgG (H + L) (ZB-2306) was purchased from ZSGB-Bio OriGene. Total ProteoExtract Kit (KGP250), Bradford Protein Assay Reagent Kit (KGA801), and WesternBright Sirius ECL (KGP1125) were all purchased from Nanjing KeyGEN Biotech. CO., LTD. ProteoExtract Cytosol/Mitochondria Fractionation Kit (QIA88) was purchased from Merck. Polyvinylidene difluoride (PVDF) membranes was purchased from Millipore Corp. Equipment utilized include Protein Measuring Instrument (Eppendorf, Germany), High Speed Refrigerated Centrifuge (Sigma, America), Instantaneous centrifuge (Kylin-bell) and Micropipette (Eppendorf, Germany).

### Establishment of a chronic poisoning model of *O. kansuensis* in SD ra

Experiments were performed in male and female SD rats (200-220 g total body weight). After a week-long adaptation period in a room with controlled temperature (21 ± 1°C) and lighting (12 h light/12 h dark), 24 SD rats were assigned to either a control group (complete feed) or experimental groups I (15% *O. kansuensis* containing 0.03‰ SW), II (30% *O. kansuensis* containing 0.06‰ SW), or III (45% *O. kansuensis* containing 0.09‰ SW), and 6 SD rats of each group were feed in 3 cages separately. One hundred and nineteen days after poisoning, and all rats showed neurological disorders at different degrees, which were considered to be successful established a chronic poisoning model of *O. kansuensis*. all rats were anesthetized with ether vapor and sacrificed by decapitation. The blood of all rats were collected and analyzed on concentration of SW in serum by HPLC (The data were not shown). The cerebrum from each animal was collected and preserved in liquid nitrogen.

### Western blot analysis

Total protein was collected by Total ProteoExtract Kit (KGP250). Isolation and extraction of mitochondria/cytosol protein was performed using the ProteoExtract Cytosol/Mitochondria Fractionation Kit (QIA88). Protein concentrations were measured using the Bradford Protein Assay Reagent Kit (KGA801). Equivalent amounts of protein were loaded separated by 12% sodium dodecyl sulfate-polyacrylamide gel electrophoresis (SDS-PAGE) at 120 V for 90 min. Proteins were subsequently transferred to PVDF membranes at 200 mA for 45 min. PVDF membranes were first blocked with 5% nonfat dry milk at room temperature for 2 h, then incubated with primary antibodies overnight at 4°C, and finally probed by HRP-conjugated secondary antibodies at room temperature for 2 h. The signal was detected using ECL reagent. Quantification was performed by Image system (Bio-Rad) from three independent experiments and analyzed with Quantity One (Bio-Rad).

### Statistical analysis

Results are expressed as mean ± standard deviation. All data were analyzed in SPASS 18.0 using one-way analysis of variance (ANOVA) followed by Duncan’s test for multiple comparisons. *P* < 0.05 was considered significant.

## Abbreviations

SD: Sprague-Dawley; SW: Swainsonine; PVDF: Polyvinylidene difluoride; SDS-PAGE: Hodium dodecyl sulfate-polyacrylamide gel electrophoresis; FADD: Fas-associated death domain; HRP: Horseradish peroxidase; ECL: Electro chemi luminescence; TNFαR: Tumor necrosis factor alpha receptor; DR3: Death recptor 3; DR4: Death recptor 4; DR5: Death recptor 5; AIF: Apoptosis inducing factor.

## Competing interests

The authors declare that they have no competing interests.

## Authors’ contributions

LH conceived the study, carried out the animal experiment, participated in western blot analysis and the statistical analysis and drafted the manuscript. ZL participated in western blot analysis and helped to draft the manuscript. WSS and WWL participated in the animal experiment. ZBY participated in the design of the study and supervised the animal experiment. All authors read and approved the final manuscript.

## References

[B1] Taylor JB Strickland JR Appearance and disappearance of swainsonine in serum and milk of lactating ruminants with nursing young following a single dose exposure to swainsonine (locoweed: Oxytropis sericea) J Anim Sci 2002 9 2476 2484 12350025 10.2527/2002.8092476x

[B2] Martyn A Tyler J Offord C McConchie R Swainsona sejuncta: a species of ornamental promise or a potential weed? Aust J Exp Agric 2003 9 1369 1381 10.1071/EA02102

[B3] Smith GS Allred KW Kiehl DE Swainsonine content of New Mexican locoweeds Proc West Sect Am Soc Anim Sci 1992 9 405 407

[B4] Medeiros RMT Barbosa RC Riet-Correa F Lima EF Tabosa IM Barros SS Gardner DR Molyneux RJ Tremorgenic syndrome in goats caused by Ipomoea asarifolia in Northeastern Brazil Toxicon 2003 9 933 935 10.1016/S0041-0101(03)00044-8 12782095

[B5] Cao GR Li SJ Duan DX Molyneux RJ James LF Wang K Tong C Cao GR, Li SJ, Duan DX, Molyneux RJ, James LF, Wang K, Tong C The toxic principle of Chinese locoweeds (*Oxytropis* and *Astragalus*): toxicity in goats Poisonous plants, Proceedings of the Third International Symposium 1992 Ames: Iowa State University Press

[B6] Zhang MS Gao QD Hou HD Li O Chen JM Zhu XW *Oxytropis kansuensis* poisoning Acta Vet et Zootech Sinic 1981 9 145 150

[B7] Wu D Liang B Shi YP Wang JH Studies on the *Oxytropis kansuensis* Bunge China Herbivores 2003 9 37 39

[B8] James LF Syndromes of locoweed poisoning in livestock Clin Toxicol 1972 9 567 573 10.3109/15563657208991031 4674446

[B9] Konstanze HP Fracis DG Neurotoxic mycotoxins: a review of fungal toxins that cause neurological disease in large animals J Vet Intern Med 1994 9 49 54 10.1111/j.1939-1676.1994.tb03195.x 8176663

[B10] Graham D Creamer R Cook D Stegelmeier B Welch K Pfister J Panter K Cibils A Ralphs M Encinias M McDaniel K Thompson D Gardner K Solutions to locoweed poisoning in New Mexico and the western united states Rangelands 2009 9 3 8

[B11] Colegate S Dorling P Huxtable C A spectroscopic investigation of swainsonine: an α-mannosidase inhibitor isolated from *Swainsona canescen* Aust J Chem 1979 9 2257 2264 10.1071/CH9792257

[B12] James LF Elein AD Molyneux RJ Warren CD Toxic species of the plant genus swainsona. In swainsonine and related Glycosidase inhibitor 1989 Ames: Iowa State Univ press

[B13] Dantas AFM Riet-Correa F Gardner DR Medeiros RMT Barros SS Anjos BL Lucena RB Swainsonine-induced lysosomal storage disease in goats caused by the ingestion of Turbina cordata in Northeastern Brazil Toxicon 2007 9 111 116 10.1016/j.toxicon.2006.08.012 17030054

[B14] Fábio M Raquel FA Joaquim EN Sílvio F Renata GSD Fabiana B David D Dale RG Franklin RC Edson MC Alpha-mannosidosis in goats caused by the swainsonine-containing plant Ipomoea verbascoidea J Vet Diagn Invest 2012 9 90 95 10.1177/1040638711425948 22362938

[B15] Fadeel B Orrenius S Apoptosis: a basic biological phenomenon with wide-ranging implications in human disease J Int Med 2005 9 479 517 10.1111/j.1365-2796.2005.01570.x 16313474

[B16] Ghobrial IM Witzig TE Adjei AA Targeting apoptosis pathways in cancer therapy CA-Cancer J Clin 2005 9 178 94 10.3322/canjclin.55.3.178 15890640

[B17] Sun JY Zhu MZ Wang SW Miao S Xie YH Inhibition of the growth of human gastric carcinoma in vivo and in vitro by swainsonine Phytomedicine 2007 9 353 359 10.1016/j.phymed.2006.08.003 17097281

[B18] Sun JY Yang H Miao S Li JP Wang SW Suppressive effects of swainsonine on C6 glioma cell in vitro and in vivo Phytomedicine 2009 9 1070 1074 10.1016/j.phymed.2009.02.012 19427771

[B19] Li ZC Xu XG Huang Y Ding L Wang ZS Yu GH Xu D Li W Tong DW Swainsonine activates mitochondria-mediated apoptotic pathway in human lung cancer A549 cells and retards the growth of lung cancer xenografts Int J Biol Sci 2012 9 394 405 22393311 10.7150/ijbs.3882 PMC3291856

[B20] Susan EL Seamus JM Caspase activation cascades in apoptosis Biochem Soc T 2008 9 1 9 10.1042/BST0360001 18208375

[B21] Kent RVK Lynn FJ Pathology of Locoweed poisoning in sheep Vet Pathol 1969 9 413 423 10.1177/030098586900600505 5371122

[B22] Colodel EM Gardner DR Zlotowski P Driemeier D Identification of swainsonine as a glycoside inhibitor responsible for Sida carpinifolia poisoning Vet Hum Toxicol 2002 9 177 178 12046976

[B23] Anglade P Vyas S Javoy-Agid F Herrero MT Michel PP Marquez J Mouatt-Prigent A Ruberg M Hirsch EC Agid Y Apoptosis and autophagy in nigral neurons of patients with Parkinson's disease Histol Histopathol 1997 9 25 31 9046040

[B24] Su JH Anderson AJ Cummings BJ Cotman CW Immunohistochemical evidence for apoptosis in Alzheimer's disease Neuroreport 1994 9 2529 2533 10.1097/00001756-199412000-00031 7696596

[B25] Yuan JY Bruce AY Apoptosis in the nervous system Nature 2000 9 802 809 10.1038/35037739 11048732

[B26] Angelos T Heart muscle and apoptosis Cardiomyopathies-From Basic Res to Clin Manage 2011 9 185 199

[B27] Adams JM Cory S The Bcl-2 protein family: arbiters of cell survival Science 1998 9 1322 1326 9735050 10.1126/science.281.5381.1322

[B28] Antonsson B Martinou JG The Bcl-2 protein family Exp Cell Res 2000 9 50 57 10.1006/excr.2000.4839 10739651

[B29] Gross A McDonnell JM Korsmeyer SJ Bcl-2 family members and the mitochondria in apoptosis Gene Dev 1999 9 1899 1911 10.1101/gad.13.15.1899 10444588

[B30] Liu P Cong GZ Du JZ Shao JJ Lin T Chang HR Studying progress of cell apoptotic pathway Hubei Agri Sci 2010 9 715 717

[B31] Inthrani RI Grégory T Shazib P Catherine B Recent advances in apoptosis, mitochondria and drug resistance in cancer cells BBA-Bioenergetics 1807 9 735 745 10.1016/j.bbabio.2011.03.010 21453675

[B32] Nika ND Stanley JK Cell death: critical control points Cell 2004 9 205 219 10.1016/S0092-8674(04)00046-7 14744432

[B33] Michael OH The biochemistry of apoptosis Nature 2000 9 770 776 10.1038/35037710 11048727

[B34] Patrice XP Santos AS Naoufal Z Bernard M Guido K Mitochondria and programmed cell death: back to the future FEBS Lett 1996 9 7 13 10.1016/0014-5793(96)00988-X 8906857

[B35] Dirk B Tak WM Mitochondrial cell death effectors Curr Opin Cell Biol 2009 9 871 877 10.1016/j.ceb.2009.09.004 19822411

[B36] Lu H Wang SS Zhao BY Isolation and identification of swainsonine from *Oxytropis glabra* and its pathological lesions to SD rats Asian J Anim Vet Adv 2012 9 822 831 10.3923/ajava.2012.822.831

[B37] Sun YF Bai DC Zhang WH Laboratory Animal Science 1998 Zhengzhou: Zhengzhou Univ Press

[B38] Shi ZC Importent Poisonous Plants of China Grassland 1997 Beijing: China Agr Press

